# Use of Observational Learning to Promote Motor Skill Learning in Physical Education: A Systematic Review

**DOI:** 10.3390/ijerph191610109

**Published:** 2022-08-16

**Authors:** Yankun Han, Syed Kamaruzaman Bin Syed Ali, Lifu Ji

**Affiliations:** 1 Faculty of Education, University of Malaya, Kuala Lumpur 50603, Malaysia; 2 Faculty of Sport and Science, Hoseo University, Asan 31066, Korea

**Keywords:** modeling, observational learning, students, physical education, motor skill learning

## Abstract

Observational learning is an effective pedagogical approach that can influence students’ motor skill development at every level of physical education (PE). This study aimed to systematically summarize the evidence on observational learning for motor skill learning in PE and to generalize the evidence on the effect of model formats and verbal cues during observational learning. An electronic search of eight databases was conducted. Eighteen studies were included and their methodological quality was assessed using the Physiotherapy Evidence Database Scale. Best evidence synthesis (BES) was used to assess levels of evidence. Strong evidence supported the effect of observational learning on students’ motor skill learning compared to students who did not participate in observational learning. Moderate evidence suggested that there is no significant difference in the effectiveness of observing an expert model compared to a self-model. Conflicting evidence was identified for the effect of the presence of verbal cues compared to the absence of verbal cues during observational learning. The results suggest that observational learning is useful for students’ motor skill learning in PE. Given the influences of potential factors, we recommend that future studies investigate how observational learning interacts with verbal cues on students’ motor skill learning.

## 1. Introduction

Physical education (PE) is an integral part of the educational system in many nations around the world [1]. One of the main goals of PE is to promote students’ motor skills [2]. Motor skill learning in PE can be defined as progress in students’ ability to demonstrate a skill, which has to be determined by a relatively sustained enhancement in implementation [3]. Maximizing the quality of the students’ motor skill learning is an initial goal of PE instruction [4]. The quality of motor skills mastered by students can be referred to as motor skill competence [5]. The development of motor skill competence enables physical activity (PA) to improve dynamically [6]. Children and adolescents with higher motor skill competence tend to pay more attention to being involved in moderate-to-vigorous physical activity (MVPA) than peers with lower motor skill proficiency [7,8]. Given the potential benefits of teaching motor skills to children and adolescents to improve PA and MVPA, it is critical that PE teachers employ effective instructional techniques to enhance students’ motor skills.

The process of learning motor skills is usually based on a theoretical framework of information processing [9]. The change in motor performance reflecting learning is facilitated by information [9]. For the initiation of motor skill learning, feedback as information is a crucial influencing element [10]. Since information is fed back from external sources, it is extrinsic in nature [11]. The temporal and spatial information of the movement is symbolically encoded by the learner’s perception and extracted and modified as needed [12,13]. This process parameterizes the movement representations. A technical model is used for demonstration, and visual or verbal cues help decrease errors [11]. One of the fundamental instructional techniques that influences motor skill learning in PE is observational learning. Observational learning also refers to modeling, which is defined as the process by which an observer attempts to replicate a demonstrated behavior or movement [14,15]. The principle of observational learning is that the observer uses the acquired cognitive representation to guide the subsequent execution of the motor skills [12,14]. According to social learning theory, observational learning influences the acquisition of a motor skill through four subprocesses, namely, attention, retention, reproduction, and motivation. The four basic components are four sequential and interrelated parameters for observational learning [12]. Attention refers to the behaviors to which the learner must pay attention when observing. It determines what type of demonstration prototype the learner selects. Retention means that the observed behaviors are stored in the memory in the form of symbols through schemata and verbalization. Reproduction means that the stored information or observed and learned behaviors are reproduced. Motivation is the demonstrative behavior that learners show out of a need under certain situational conditions. According to Bandura, the process of motor learning involves the establishment of conceptual representations. Through the constant transformation of observed behaviors, representations are gradually transformed into symbolic codes that enable the storage of observed actions [16]. If observers can transform the conceptual representations into symbolic codes and perform cognitive rehearsal, then reproduction will be more accurate [17]. Therefore, attention and retention play a crucial role in the acquisition phase of motor skill learning. If the conceptual representations and execution do not match, it is impossible for the observer to accurately recall the symbolic code when reproducing actions, especially in complex actions [16]. Therefore, reproduction and motivation are useful for reproducing motor performance. This is because discrepancies between cognitive representations and execution are difficult to detect [16]. Therefore, it is crucial that the observer reproduces the modeled motor skills with motivation and motoric capability [14]. Observational learning has been a hot topic in the field of motor skill learning for decades [18] and is regarded as one of the most powerful methods for learning motor skills [12,19,20]. In addition, research on PE suggests that observational learning is an effective pedagogical tool that influences students’ learning of motor skills [21,22]. However, conclusions about the effect of observational learning on students’ motor skill learning in PE settings are divided.

Previous studies have reported that observational learning is more effective in motor skill learning than not participating in observational learning in PE. For example, Potdevin et al. (2018) found that in PE classes for middle school students, their classmates significantly improved basic gymnastics skills compared to those who did not use observational learning [21]. Kretschmann (2017) reported that the presence or absence of observational learning in swimming lessons for high school students resulted in a large difference in students’ swimming skills learning [23]. Other studies in college and elementary school PE found that the availability of the observational model significantly improved students’ motor skills in badminton [24] and basketball [25]. However, there was evidence that observational learning does not have a significant impact on students’ motor skill learning [26,27]. Recent studies indicated that observational learning does not cause significant differences in student performance in the shot put [26] and standing long jump [27]. One of the reasons for this inconsistent result is the observational model’s skill level [28,29]. Giannousi et al. (2017) pointed out that whether the model they observe is expert or non-expert is a key factor in students’ motor skill learning [30]. Some studies suggested that observing expert models in PE is more conducive to motor skill learning [24,27,31,32]. In contrast, some studies were negative about the role of expert models in PE and suggested that non-expert models can more actively promote the learning of sports skills in gymnastics [21], swimming [23,30], and soccer [33]. 

In addition, verbal cues play an important role in observational learning [28]. Teacher verbal cues are all the information that the teacher verbally expresses about students’ learning of motor skills in PE classes [34]. Relevant concepts of attention and information processing in motor skill acquisition provide a theoretical basis for verbal cues [35]. Landin (1994) outlines two main features of attention that theoretically support the use of verbal cues in motor skill acquisition, namely attentional focus and attentional transition [35]. On the one hand, attentional focus can vary in different dimensions: broad, narrow, internal, and external along the breadth and directional continuity of each moment. On the other hand, attention must shift from one dimension to another depending on the situation when learning a motor skill. Verbal cues can effectively help learners to acquire appropriate attentional patterns and assist them in transitions between different patterns [36]. In addition, information processing facilitates the identification and retrieval of relevant information [35]. The three functions of information processing, namely perceptual processing, decision processing, and effector processing, facilitate motor performance [37]. Traditionally, verbal cues have been considered a useful pedagogical strategy in PE instruction to enhance motor learning [38]. This is because verbal cues can complement the visual teaching of specific technical movements that students ignore in PE instruction [35]. However, some studies have found that the absence of verbal cues in PE observational learning can significantly improve students’ skills in swimming [23] and badminton [24]. The inconsistency of the different research findings indicates that more research is needed on the influence of verbal cues in observational learning on students’ motor skill learning in PE.

Although experts believe that information gained during observational learning is conducive to motor skill learning [39,40], current research on the findings of observational learning on students’ motor skill learning is inconsistent [30,33]. The ideal way to make comprehensive recommendations is to conduct a systematic review of the existing literature. Therefore, this study aims to systematically review the existing published studies on the effects of observational learning on students’ motor skill learning in PE to clarify the aforementioned issues. Specifically, this study begins with a summary of the evidence on observational learning, followed by an observation of the model formats and use of verbal cues during observational learning on students’ motor skill learning in PE lessons. Based on social learning theory and the characteristics of the included articles, this review defines observational learning as a teaching method in which PE teachers use role models to help students build the cognitive representation of motor skills and then guide students to replicate and reproduce the motor skills demonstrated in school physical education. The results of this systematic review may help PE teachers confirm the role of observational learning in promoting students’ motor skill learning in school physical education, clarify the differential influence of model types on motor skill learning, and avoid confounding factors that influence verbal cues in observational learning.

## 2. Materials and Methods

The method of this systematic review followed the Preferred Reporting Items for Systematic Reviews and Meta-Analysis guidelines (PRISMA) [41].

### 2.1. Search Strategy

A systematic search for relevant articles published on the topic of observational learning in PE was conducted in the electronic databases of Scopus, Web of Science, EBSCOHost (including Education Research Complete, Academic Search Elite, ERIC, MEDLINE, Psychology and Behavioral Sciences Collection, SPORTDiscus). The following various combinations of key terms were used (1) “observational learning” OR “learning by observation” OR “model learning” OR “video feedback” OR “vicarious learning” OR “demonstration” OR “visual feedback” OR “observation” (2) “motor skill” OR “sports skill” OR “motor performance” OR “motor learning” OR “skill learning” OR “skill acquisition” OR “athletic skill” OR “basic skill” OR “fundamental skill” (3) “physical education” OR “school sports” OR “PE” OR “student*” OR “college*” OR “university*” OR “school*”. A systematic search, for potentially eligible literature published before June 2022, was conducted. The search strategy used for each database is shown in Table 1. A manual search was performed for potential articles in the reference list of included studies.

### 2.2. Inclusion and Exclusion Criteria

Articles that meet the following criteria will be included in this systematic review: (1) Studies must apply observational learning to PE in schools and focus on changing the level of students’ motor skill learning. Studies that used observational learning for teaching or training in other scenarios were excluded. (2) Studies must be empirical and observational learning is a prerequisite operating variable. Other studies were excluded. (3) The subjects must be ordinary students in schools, including elementary school, middle school, high school, and ordinary university. Those who specialized in teaching and training in physical education in schools were excluded. (4) Students participating in the study must be healthy subjects; subjects with disabilities or other medical conditions were excluded. (5) Published peer-reviewed journals with full text in English were included; other types of gray literature, including dissertations, theses, reviews, conference proceedings, and unpublished articles were excluded.

### 2.3. Data Extraction

Data were extracted from each study using a predesigned table containing the following information: (1) first author’s name; (2) publication year; (3) nations; (4) student characteristics, including school type, sample size, age, and skill level; (5) study design; (6) observational learning format and instructional strategy; (7) discipline and sport skill; (8) duration of instruction; (9) main outcomes.

### 2.4. Methodological Quality Assessment

The methodological quality of the included studies was assessed using the Physiotherapy Evidence Database (PEDro) Scale [42]. The PEDro scale, based on the Delphi consensus technique [43] and developed to assess the methodological quality of studies, consists of 10 items, namely random allocation, concealed allocation, baseline comparability, blinded students, blinded teachers, blinded assessors, adequate follow-up, intention-to-treat analysis, comparisons between groups, and point estimates and variability [42]. The scale has two possible options, 0 or 1. In the scale, 0 indicates that the assessment item is missing, and 1 indicates that the assessment item is present. Scores range from 0 to 10, with higher scores indicating better quality of the research method used to evaluate the article. Because there are no published, validated cutoffs for the PEDro scale, researchers generally use a PEDro score of 5 or higher as high quality and vice versa as low quality [44]. The reliability of the PEDro scale was tested and found to have an intraclass correlation coefficient (ICC) of 0.56 (95% CI = 0.47–0.65) for ratings by individuals and an ICC for consensus ratings of 0.68 (95% CI = 0.57–0.76) [42]. This scale has been used in recent similar systematic reviews and has been evaluated as an effective tool for assessing the methodological quality of motor skill learning studies [11,45,46].

### 2.5. Evidence Syntheses

Because of the heterogeneity of the included studies, such as inconsistencies in data reporting, a meta-analysis could not be performed. Best evidence synthesis (BES) is an intelligent alternative to a meta-analysis [47,48] when studies included in the systematic review cannot establish sufficient weight [44], which has been applied to other similar systematic reviews [45,49]. The strength of this rating system is that it uses methodological quality, several studies, and consistency of results as the main indicators for evaluation [47] and prioritizes quality over quantity [50]. Although five levels of evidence were used to rank studies in this grading system, we decided to summarize the evidence according to the following rules: (1) Strong evidence, supported by consistent results from 3 or more high-quality studies. (2) Moderate evidence, supported by consistent results from 2 high-quality studies. (3) Limited evidence, supported by consistent results from 1 high-quality report. (4) Conflicting evidence, results of studies were inconsistent. (5) No evidence, only low-quality studies reported consistent results [51].

### 2.6. Reliability of Systematic Review Procedures

Two authors involved in this study (YKH and LFJ) performed each of the above procedures independently. Disagreements between the two authors on the results of a session were resolved by thorough discussion. If the two authors could not agree, a third author was consulted until a consensus was reached on all results.

## 3. Results

### 3.1. Literature Search

**Figure 1:**
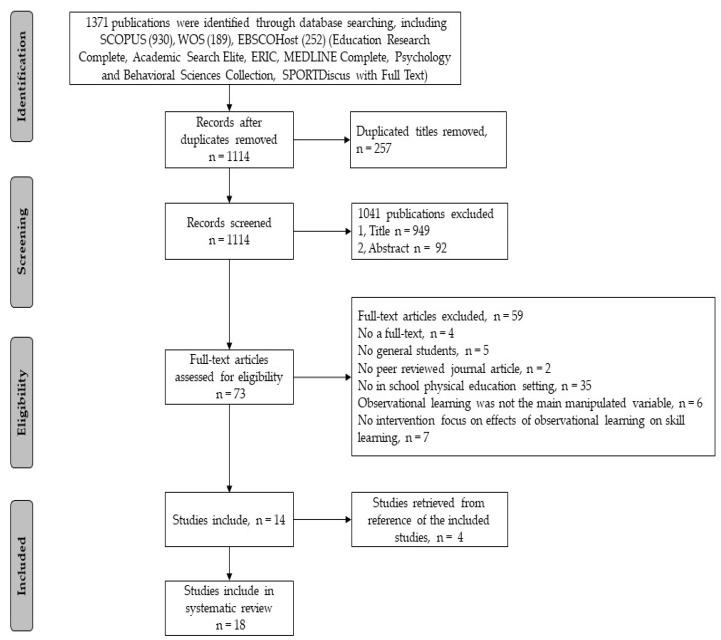
PRISMA Flowchart of The Literature Search.

Figure 1 shows the process of study selection. A total of 1371 articles were retrieved from the predefined database. Duplicate titles were excluded, and titles and abstracts were identified for 1114 articles. However, 1041 articles that did not meet the criteria were further excluded. A total of 73 potentially relevant articles were assessed, 14 of which fully met the criteria for inclusion in the systematic review. After a manual search of the reference lists of included articles, four articles that met the criteria were included in the scope of this review. Therefore, 18 articles were finally included in this systematic review.

### 3.2. Methodological Quality

As shown in Table 2, the eighteen articles included in the present review had PEDro scores between two and seven, with a median and mode of five. Twelve articles had a score of five or more, while the remaining six were below five. The majority of articles (nine) were scored five, accounting for 50% of all articles in the current study. The remaining articles were scored two, four, and six (two each). According to the evaluation rules (see Section 2.4), twelve articles with a rating of five or more are high-quality articles, and the remaining six are low-quality articles. The evaluation criterion that all included articles met the most was “comparison between groups.” The weakest methodological quality was “blind” and “concealed allocation.” Only one article used the “single-blind” technique for teachers and students, and two articles used the “concealed allocation” technique.

### 3.3. Study Characteristics

Table 3 shows the characteristics of the 18 articles included in this systematic review. The articles were published between 1988 and 2022. Nine articles were published in recent decades, accounting for 50% of the studies included in the current review. The researchers were from ten countries and regions, six of which were from the United States, followed by Greece and France with three and two, respectively. The remaining countries and regions each had one, including Ireland, Spain, United Kingdom, Germany, Netherlands, Italy, China, and Taiwan. Students involved in the study came from a variety of schools, from elementary to college. Four of the study participants were from elementary and middle schools, with an average age of about 6–12 years and 13–14 years. Five study participants were from high school or college, with an average age of about 15–18 years and 18-27 years. Boys and girls participated in seven studies, with most studies having relatively equal numbers of participants of both genders. Five studies included only girls and two included only boys. Four studies did not report information on the gender of the participating students.

Sample sizes can be divided into three segments: Four studies with samples of more than 100 students, six with samples between 50 and 100 students, and eight with fewer than 50 students. The skill level of the students was mainly novice (11 studies), the students in two studies were experienced, and the remaining studies did not provide any information. All studies were pretest and post-test designs, except one, which was a mixed-methods design. Instructional content included a variety of disciplines and motor skills. Three studies focused on swimming, two studies focused on volleyball, and one each focused on juggling, basketball, hurdling, soccer, badminton, gymnastics, shot put, and standing long jump. The duration of the interventions varied, ranging from 5 months to 8-min training sessions.

### 3.4. Effects of Observational Learning in Physical Education

Although various forms of observational learning models were used in the 18 studies included in this systematic review, they can be broadly divided into observational expert models and non-expert models. The non-expert models include the self-model, the peer model, the novice model, the unskilled model, the coping model, and the learning model. The instructional strategies used for observational learning were mostly verbal cues. Based on the above, we propose three comparison groups and assign the 18 included studies to these groups to summarize the available evidence that observational learning improves motor skill learning in PE. If a study contains multiple group comparisons, the analysis is repeated with different comparison groups. Comparisons of less than three articles were not generalized as available evidence based on previous experience [45].

#### 3.4.1. Effects of the Present Verse Absence of Observational Learning on Students’ Motor Skill Learning

Fourteen of the 18 included studies compared the effects of student participation in observational learning versus the absence of observational learning in PE. Eleven studies reported that observational learning improved students’ motor skills in the sports of Bachman ladder-climbing [14], gymnastics [21], swimming [23,30], badminton [24], basketball [25], precision ball throwing [27], soccer [33], golf [53], dart-throwing [54], hurdling [57]. In contrast, three studies found that observational learning did not significantly improve students’ motor skills compared to lack of observational learning, including shot put [26], tennis [52], and volleyball [56]. In addition, Experiment 2 of the study by Sorgente et al. (2022) reported that the use of observational learning in the experimental group did not significantly improve students’ performance in standing long jump compared to non-observational learning in the control group [27]. Six of 11 studies reported that observational learning effectively improved the motor skills of students with a PEDro score of five [21,23,24,27,30,53]. Following the rules of evidence synthesis (see Section 2.5), it is suggested that there is strong evidence that the use of observational learning is more effective in promoting students’ motor skill learning than observational learning which is not available in PE.

#### 3.4.2. Effects of Observing Expert Models Versus Non-Expert Models on Students’ Motor Skill Learning

Nine of the 18 included studies compared the effects of student observation of expert models versus non-expert models on student learning of motor skills in PE. Four studies compared the effects of student observation of expert models and self-models on student learning of motor skills. Two studies found no significant difference in motor skill learning between the two models [15,52]. Zetou et al. (2002) reported that the expert model was more effective than the self-observation model in improving students’ volleyball skills [31]. In contrast, Giannousi et al. (2017) compared the effects of the observational self-model and the expert model on students’ swimming skills learning, and the results showed that the self-model significantly improved students’ movement skills [30]. Lirgg et al. (1991) compared a model with experienced teachers and peers to a model with unskilled teachers and peers for learning Bachman ladder climbing skills [14]. Results showed that the motor skills of students who received the skilled model were significantly better than those of students who received the unskilled model. D’Arripe-Longueville et al. (2002) investigated the expert model and the novice model for students learning the breaststroke turn. The group that followed the expert model showed better swimming skills [32]. However, Kitsantas et al. (2000) found that the coping model was more effective than the expert model in helping students learn dart-throwing techniques [54]. Meaney et al. (2005) found no significant difference between the skilled model and the learning model in helping students juggle scarves [55]. Barzouka et al. (2007) compared the effect of the expert model and the combination model (expert model and self-model) in supporting students’ learning of volleyball skills [56]. The results showed that there was no significant difference between the two models. In this series of comparisons, only four studies compared the observational expert model and the self-model. Two studies reported no significant differences between the two models in student motor skill development [15,52]. The two studies had PEDro values of six and five, respectively. Following the rules of evidence synthesis (see Section 2.5), it is suggested that moderate evidence suggests that there is no significant difference between observing the expert model and the self-model in students’ motor skill learning in PE lessons.

#### 3.4.3. Effects of the Present Verse Absence of Verbal Cues on Students’ Motor Skills Learning

Seven studies compared the presence and absence of verbal cues during observational learning. Three studies showed that the presence of verbal cues significantly improved students’ motor skills compared to the absence of verbal cues in gymnastics [21], basketball [25], and freestyle swimming [30]. In contrast, three studies indicated that the absence of verbal cues was more effective than the presentation of verbal cues in improving front crawl swimming [23], badminton [24], and golf swing [53]. In the remaining study, no significant difference was found between the presence and absence of verbal cues during observational learning in motor skill learning [15]. In comparison to the absence of verbal cues during observational learning, conflicting evidence was found for the effects of the presence of verbal cues (50%, 3/6 of studies) on students’ motor skill learning.

## 4. Discussion

This systematic review aimed to generate evidence of the role of observational learning in promoting students’ motor skills in PE. Based on the different models of observational learning and verbal-cue-based instructional strategy used in the 18 included studies, we proposed three comparison groups to synthesize the precise evidence. Specifically, (1) effects of the present verse absence of observational learning on students’ motor skill learning. (2) effects of expert model verse non-expert model on students’ motor skill learning. (3) effects of the present verse absence of verbal cues on students’ motor skill learning.

### 4.1. Effects of the Present Verse Absence of Observational Learning on Students’ Motor Skill Learning

Our results suggest that the use of observational learning is more effective than the absence of observational learning in promoting students’ motor skill learning. According to BES, this result was supported by strong evidence. In general, observational modeling can be useful in promoting the development of motor skills [28]. This is consistent with our findings in this review. In this systematic review, 14 studies compared the effects of observational learning in students versus non-observational learning on motor skill learning in PE. Eleven studies (78.6%, 11/14) confirmed that observational learning can be effective in improving students’ motor skills. Previous studies have also shown the remarkable results of model observation on learning motor skills in the acquisition phase [58,59,60,61,62]. For example, students with observational learning improved their performance in complex gymnastics skills in the acquisition phase [59]. In addition, a group of college students improved their performance in stacking cups after observing a model in the acquisition phase [58]. According to social learning theory, the four components that enable observational learning constructs a process of cognitive adaptation in motor skill acquisition [13]. During this process, movements are organized into cognitive representations at the cognitive level and then developed into symbolic codes to guide execution. The richer the cognitive representation, the easier it is for the observer to demonstrate the observed motor skill [16]. Therefore, students who learn through observation in PE tend to show better motor skills in the acquisition phase than those who do not participate in observational learning [21,23,24,30]. However, some studies have found that there is no significant difference in student motor skill performance in the acquisition phase, whether or not observational learning is provided [26,56]. Numerous factors, such as students’ experience, gender, and age, play a critical role in the process of observational learning [28]. For example, the cognitive representational ability of adults is three times that of fourth and fifth-grade elementary students [22]. If observers cannot effectively convert modeled actions into symbolic codes and mentally rehearse them, they cannot produce the observed model [17]. However, from the three included studies, we were unable to determine what factors contributed to the lack of significant differences in students’ motor skill performance during the acquisition phase, regardless of whether observational learning was present or not. Future research interventions should address this issue.

### 4.2. Effects of Expert Model Versus Non-Expert Model on Students’ Motor Skill Learning

Comparisons of fewer than three studies were not pooled as evidence due to the experience of the previous study [45]. Therefore, in this group of comparisons, only the expert model was compared with the self-model with no less than three studies that can be summarized as evidence. The result showed that the observation of the expert model and the self-model in PE did not differ significantly in students’ learning of motor skills, but the level of evidence for this result was moderate. This result is consistent with the previous study that provided limited evidence of the effects of the observational expert model compared with the self-model on student motor performance [45].

However, the existing literature maintains controversial arguments about the effects of the expert model and the self-model in motor skill learning. In general, studies have concluded that the expert model is more effective in promoting observer learning of motor skills [11,28,45]. Expert modeling has been defined as a demonstration performed by elite performers [63]. Barzouka et al. (2007) suggested that the expert performs the technical movements correctly and the perfect presentation is more likely to encourage students to imitate it and perform better [56]. Therefore, when students observe the expert model, they are more likely to adopt the professional technical parameters of the movements. For example, after observing an expert model, a group of children significantly improved their basic volleyball skills compared to the children who modeled themselves [31]. In one of their experiments, Sorgente et al. (2022) found that elementary school beginners improved their accurate throwing of the ball while watching the traces of the expert model [27]. In addition, the expert model was very helpful for school beginners in performing the breaststroke turn in swimming [32].

This contrasts with self-modeling, in which learners observe their movement execution [11]. Self-modeling usually focuses on correcting errors in motor performance but can also be useful as a learning model [15]. Previous studies have shown that the effect of the model on motor skill learning is influenced by the skill level of the observer. [21,23,64,65]. In this review, 11 of the 18 studies included novices in the observation of motor skill modeling. Bandura noted that novices may feel that the expert model’s demonstration of movements is beyond their abilities and therefore they are unable to fully imitate them [66]. According to social learning theory, while learning a motor skill, learners need to observe their performance to know how well they have mastered the skill, how much effort they need to put in, and when they can correctly assess the learning strategy [12]. On the other hand, novice learners gain vicarious experience by observing similar models to promote skill learning [67]. When observing expert models, they cannot experience this similarity [68]. Therefore, viewing a self-model could maximize the similarity between the model and observer and perform the modeled skills [60,69]. For example, significant improvement in acquisition phase performance was found after a group of middle school students observed self-modeling while performing gymnastics skills [21]. In addition, self-modeling significantly improved elementary students’ freestyle swimming skills after the intervention [30]. Similarly, the self-modeling group was found to perform better in crawl swimming during the acquisition phase [23]. This is confirmed by another study that found that students in the self-modeling group performed better in soccer [33].

Based on the above details, although the result of this study is moderate evidence for the observation that the expert model versus the non-expert model (self-model) does not show a significant difference in terms of impact on students’ motor skill learning, it should be explained with caution and other factors that may influence the use of modeling in motor skill learning should be considered. These include task complexity, participant age, and skill type (i.e., open or closed, continuous, serial, or discrete) [68]. Future studies should focus on these elements to address this issue.

### 4.3. Effects of the Present Verse Absence of Verbal Cues on Students’ Motor Skill Learning

Verbal cues also called verbal feedback or verbal instruction [35], are defined as any information verbally expressed by the teacher in PE classes about students’ learning of motor skills [34]. Traditionally, verbal cues have been considered a useful pedagogical strategy in PE classes to enhance motor learning [38]. This is because verbal cues can supplement the visual teaching of specific technical movements that students ignore in PE classes [35]. However, in this review, we found conflicting evidence on the effects of the presence of verbal cues on students’ motor skill learning compared to the absence of verbal cues in observational learning in PE. Although this result was not expected, it is not surprising given that the use of verbal cues in PE is confounded by many factors, including task type, initial student ability, student cognitive level, and characteristics of verbal cues. The use of modeling in conjunction with verbal cues is a commonly used strategy in PE to improve students’ motor skills, for example, in gymnastics [21], basketball [25], and swimming [30]. However, if the above confounding factors cannot be adequately addressed, it may lead to an unsatisfactory effect on motor skill learning. First, the combination of modeling and verbal cues means more feedback information [70], which potentially increases students’ burden in extracting performance-related information [71]. In addition, too many verbal cues can disrupt the rhythm of practice and impair motor skill performance [72]. Similarly, whether the verbal cues given are linguistically appropriate [73] or precise and understandable [11] also influences the effect of verbal cues on observational learning in PE. Moreover, students’ cognitive abilities vary at different ages [74,75], and verbal cues that are too detailed are not appropriate for students in the early stages of cognition to learn motor skills [35]. Furthermore, there are different types of verbal cues, such as informational cues, corrective cues, and praise cues. Given numerous potential factors, such as task complexity and student ability, that affect the role of different forms of verbal cues in observational learning, it is difficult to generalize which verbal cues are beneficial to students’ motor skill learning [45]. A previous study also found conflicting evidence on the effects of different verbal cues on motor skill learning in PE [45]. In the context of school PE, the existing literature on combining modeling and verbal cues on students’ motor skill learning lacks focus on these confounding factors. Including the seven studies in this comparison group. At least, these studies did not examine the questions of what (characteristics of verbal cues), why (nature of tasks), and how (initial student ability, student cognitive level) of verbal cues. Future research is needed to address the issue of how verbal cues affect student motor learning in PE by addressing these confounding factors.

## 5. Limitation

We must admit that there are several limitations to this review. First, despite a thorough literature search, some relevant studies may have been overlooked because the keywords used in this review were limited. Second, we did not summarize evidence on motor skill learning in the retention phase. The retention phase is considered the extent to which motor skills are reproduced without intervention [76]. Therefore, it was not clear to what extent motor skills could be maintained in students after the intervention of observational learning. Third, the studies included in this review lack the design of verbal cues. Therefore, it is challenging to generalize the evidence on the effects of the presence or absence of verbal cues on students’ motor skill learning during observational learning in PE.

## 6. Conclusions

This review has systematically summarized the helpful evidence that students use observational learning to influence motor skill learning in PE. The results presented showed that there is strong evidence for the effects of observational learning on students’ learning of motor skills in PE. Moderate evidence was found for the usefulness of expert model observation compared to non-expert model observation, i.e., self-modeling. A conflicting result was found for the effectiveness of verbal cues in observational learning compared to the absence of verbal cues. Future studies are needed from the perspective of better methodological design and representative sample sizes to determine the potential effects of different elements of observational learning, including task type, student characteristics, and the characteristics of verbal cues, which could support motor skill learning in PE.

## Figures and Tables

**Table 1:** Search Strategies.

Database	Outcomes	Search String
Scopus	930	(TITLE-ABS-KEY((“observationallearning”OR”learningbyobservation”OR”modellearning”OR”videofeedback”OR”vicariouslearning”OR”demonstration”OR”visualfeedback”OR”observation”))ANDTITLE-ABS-KEY((“motorskill”OR”sportsskill”OR”motorperformance”OR”motorlearning”OR”skilllearning”OR”skillacquisition”OR”athleticskill”OR”basicskill”OR”fundamentalskill”))ANDTITLE-ABS-KEY((“physicaleducation”OR”schoolsports”OR”PE”ORstudent*ORcollege*ORuniversity*ORschool*)))AND(LIMIT-TO(LANGUAGE,”English”))AND(LIMIT-TO(DOCTYPE,”ar”))
Web of Science	189	((TS = ((“observational learning” OR “learning by observation” OR “model learning” OR “video feedback” OR “vicarious learning” OR “demonstration” OR “visual feedback” OR “observation”))) AND TS = ((“motor skill” OR “sports skill” OR “motor performance” OR “motor learning” OR “skill learning” OR “skill acquisition” OR “athletic skill” OR “basic skill” OR “fundamental skill”))) AND TS = ((“physical education” OR “school sports” OR “PE” OR student* OR college* OR university* OR school*)), Peer-reviewed journal articles, English
EBSCOHost	252	((“observational learning” OR “learning by observation” OR “model learning” OR “video feedback” OR “vicarious learning” OR “demonstration” OR “visual feedback” OR “observation”)) AND ((“motor skill” OR “sports skill” OR “motor performance” OR “motor learning” OR “skill learning” OR “skill acquisition” OR “athletic skill” OR “basic skill” OR “fundamental skill”)) AND ((“physical education” OR “school sports” OR “PE” OR student* OR college* OR university* OR school*)) in Title, Abstract, Keywords. Filters: English

Note: EBSCOHost includes Education Research Complete, Academic Search Elite, ERIC, MEDLINE Complete, Psychology, and Behavioral Sciences Collection, SPORTDiscus with Full Text.

**Table 2:** PEDro Scores.

Study	Random Allocation	Concealed Allocation	Groups Similar at Baseline	Blind Student	Blinded Teacher	Blinded Assessor	Follow Up	Intention to Treat Analysis	Between Group Comparison	Point Estimates and Variability	PEDro Score
Miller (1988) [52]	1	0	1	0	0	0	1	1	1	1	6
Lirgg (1991) [14]	1	0	0	0	0	1	1	0	1	0	4
Austin (1992) [53]	1	0	0	0	1	1	1	0	1	0	5
McCullagh (1997) [15]	1	0	0	0	0	1	1	1	1	0	5
Kitsantas (2000) [54]	1	0	0	0	0	0	1	0	1	0	3
d’Arripe-Longueville (2002) [32]	0	0	0	0	0	1	1	0	1	1	4
Zetou (2002) [31]	1	0	1	0	0	0	1	0	1	1	5
Meaney (2005) [55]	1	0	0	0	0	0	1	1	1	1	5
Barzouka (2007) [56]	1	1	1	0	0	0	1	1	1	1	7
O’ Loughlin (2013) [25]	0	0	0	0	0	0	1	0	1	0	2
Palao (2013) [57]	1	0	0	0	0	0	1	0	1	0	3
Harvey (2014) [33]	0	0	0	0	0	0	1	0	1	0	2
Kretschmann (2017) [23]	1	0	1	0	0	0	1	0	1	1	5
Giannousi (2017) [30]	1	0	0	0	0	1	1	0	1	1	5
Hung (2017) [24]	1	0	1	0	0	0	1	0	1	1	5
Potdevin (2018) [21]	0	0	1	1	0	0	1	0	1	1	5
Kok (2020) [26]	0	1	1	1	0	0	1	1	1	0	6
Sorgente (2022) [27]	1	0	1	0	0	0	1	0	1	1	5

**Table 3:** Summary Characteristics of Included Studies.

First Author (Year)	Characteristics of Participants					Study Design	Observational Learning Format and Instructional Strategy		Discipline and Skill	Intervention Length	Main Outcomes	
	School Type	Observer	Sample Size	Mean Age	Skill Level							
Miller (1988) USA [52]	University	University students (28 boys, 27 girls)	55 G1 19 G2 17 G3 19	Not reported	Novice	Pre-posttest	G1: no model G2: self-model G3: expert model	G1,2,3: verbal cues	Tennis forehand and backhand drive	1200 minutes	No significant differences between groups	
Lirgg (1991) USA [14]	Middle school	Middle school girl students	100 20/group	Not reported	Not reported	Control experiment	G1: expert teacher model G2: expert peer model G3: unskilled teacher model G4: unskilled peer model G5: no model	G1–5: verbal cues	Bachman ladder task	6 trial blocks (30 trials)	G1,2 had better performance than other groups. G1 is better than G2.	
Austin (1992) USA [53]	University	University students (16 boys, 4 girls)	20 EG 10 CG 10	20–27 years	Novice	Pre-posttest	EG: expert model CG: no	EG: no CG: verbal cues	Golf swing	5 weeks	EG had better golf swing performance.	
McCullagh (1997) USA [15]	University	University girl students	40 10/group	Not reported	Novice	Control experiment	G1: self-model G2: expert model G3: peer-model G4: peer-model	G1,2,3: verbal cues G4: no	free-weight squat lift	5 trials	No significant differences	
Kitsantas (2000) USA [54]	High school	High school girl students	60 10/group	14.7 years	Novice	Control experiment	EG1: coping model EG2: coping model EG3: expert model EG4: expert model EG5: no CG: no	EG1,3,5: affirmative response EG2,4 and CG: no affirmative response	Dart throwing	Not reported	The coping model had the highest dart-throwing performance. Expert model is better than no model.	
d’Arripe-Longueville (2002) France [32]	High school	High school students (24 boys, 24 girls)	48 EG 24 CG 24	18.3 years	Novice	Pre-posttest	G1: novice model G2: intermediate model G3: expert model	G1,2,3 same-gender modeling and given verbal information	Swimming breaststroke turn	8 min training session		G3 had the best skill performance. Boys skilled modeling scored the highest performance than that of boys and girls in other models.
Zetou (2002) Greece [31]	Elementary school	Elementary school students (63 boys, 53 girls)	116 G1 51 G2 64	11.7 years	Novice	Pre-posttest	G1: expert model G2: self-mode	EG, CG: verbal cues	Volleyball set and serve	8 weeks		G1 had better performance (results and form) in set skill and form in serve skill.
Meaney (2005) USA [55]	Elementary school	Elementary school girl students	40 10/group	10 years	Not reported	Mixed methods	G1: male expert model G2: female expert model G3: male learning model G4: female learning model	G1,2 adult and child demonstrate error-free. G3,4 adult and child demonstrate gradually reduced error	Juggling scarves	Not reported		No significant differences between groups
Barzouka (2007) Greece [56]	High school	High school girl students	53 EG1 18 EG2 16 CG 19	13.1 years	Novice	Pre-posttest	EG1: expert model EG2: expert and self-model CG: no	EG1, EG2, CG: verbal cues	Volleyball Reception	6 weeks		No significant differences between groups
O’ Loughlin (2013) Ireland [25]	Elementary school	Elementary school students (12 boys, 10 girls)	23	9–10 years	Not reported	Pre-posttest	G1: self-model G2: no	G1: teacher verbal inquiry G2: no	Basketball free throw, chest pass dribble, bounce pass, jump shot, and lay up	10 weeks		G1 effectively improved students’ various basketball skills.
Palao (2013) Spain [57]	High school	High school students	60 G1 17 G2 21 G3 22	15 years	Not reported	Pre-posttest	G1: no model G2: expert model and self-model G3: expert model and self-model	G1: teacher verbal cues G2: teacher verbal cues G3: peer verbal cues	Track and field, hurdle	5 lessons		G2 had significantly improvements in skill execution, and practice.
Harvey (2014) UK [33]	Middle school	Middle school boy students	34 G1 12 G2 12 G3 10	13-14years	Experienced	Pre-posttest	G1: self-model (first 3 weeks) G2: self-model (second 3 weeks) G3: no	G1,2,3: group discussion	Soccer offensive and defensive skills	6 weeks		G1,2 had better performance than G3 under modeling conditions.
Kretschmann (2017) Germany [23]	High school	High school students	31 EG 16 CG 15	Not reported	Experienced	Pre-posttest	EG: self-model CG: no	EG: no CG: verbal cues	Swimming front crawl	7 weeks		EG students significantly improved race performance of front crawl
Hung (2017) Taiwan China [24]	University	University students	225 G1 118 G2 107	Not reported	Not reported	Pre-posttest	G1: expert model and self-model G2: no	EG: no CG: verbal cues	Badminton serve, clear	5-months		G1 significantly improved badminton skills
Giannousi (2017) Greece [30]	University	University boy students	60 G1 15 G2 16 G3 14 G4 15	18.7 years	Novice	Pre-posttest	G1: self-model G2: expert model G3,4: no	G1-3: verbal cues G4: no	Freestyle swimming	7 weeks		G1 was effective in improving students’ skills.
Potdevin (2018) France [21]	Middle school	Middle school students (22 girls, 21 boys)	43 EG 18 CG 25	EG: 12.4 years CG: 12.6 years	Novice	Control experiment	EG: self-model CG: no	EG: verbal cues CG: no	Gymnastic front handstand and flat back	5 weeks		EG students significantly improved motor skills (arm-trunk angle)
Kok (2020) Netherland [26]	Middle school	Middle school students (24 boys, 32 girls)	56 EG 22 Yoked 17 CG 17	12.7 years	Novice	Pre-posttest	EG, Yoked: expert model and self-model CG: no	EG, Yoked, CG: verbal cues	Shot-put	9 weeks		No significant differences between groups
Sorgente (2022) Italy [27]	Elementary school	Elementary school students	Test1 594 G1 200 G2 195 G3 199 Test2 198 G1 66 G2 68 G3 64	6–10 years	Novice	Pre-posttest	G1,2: expert model G3: no	G1: focus on technique G2: focus on the goal G3: no	Experiment 1 Precision ball throwing, Experiment 2 Standing long Jump	Experiment 1 7 block × 3, Experiment 2 2 attempts		Experiment 1 G1 with age older students had better skill performance. Experiment 2 No significant difference.

Note: G-group; EG-experimental group; CG-control group.

## Data Availability

Not applicable.
